# The impact of cannabis legalization for recreational purposes on youth: A narrative review of the Canadian experience

**DOI:** 10.3389/fpsyt.2022.984485

**Published:** 2022-09-23

**Authors:** Dafna Sara Rubin-Kahana, Jean-François Crépault, Justin Matheson, Bernard Le Foll

**Affiliations:** ^1^Child and Youth Mental Health Division, Centre for Addiction and Mental Health, Toronto, ON, Canada; ^2^Department of Psychiatry, Faculty of Medicine, University of Toronto, Toronto, ON, Canada; ^3^Communications and Partnerships, Centre for Addiction and Mental Health, Toronto, ON, Canada; ^4^Dalla Lana School of Public Health, University of Toronto, Toronto, ON, Canada; ^5^Translational Addiction Research Laboratory, Centre for Addiction and Mental Health, Toronto, ON, Canada; ^6^Department of Pharmacology and Toxicology, University of Toronto, Toronto, ON, Canada; ^7^Departments of Family and Community Medicine, University of Toronto, Toronto, ON, Canada; ^8^Department of Psychiatry, Campbell Family Mental Health Research Institute, Centre for Addiction and Mental Health, Toronto, ON, Canada; ^9^Institute of Medical Science, University of Toronto, Toronto, ON, Canada; ^10^Waypoint Centre for Mental Health Care, Waypoint Research Institute, Penetanguishene, ON, Canada

**Keywords:** youth, adolescents, legalization, cannabis policy, Canada, cannabis (marijuana)

## Abstract

Cannabis legalization for non-medical purposes (subsequently referred to as “cannabis legalization” or “legalization”) took place in Canada in October 2018. One of the federal government's stated goals with cannabis legalization was to protect Canadian youth from cannabis-related harms. The main objective of this narrative review is to describe the impact of cannabis legalization on Canadian youth. To that end, we discuss the regulation of the Canadian cannabis market, outline changes in the epidemiology and parameters of cannabis use (modes of use, potency of cannabis) among youth, and discuss prevention and education initiatives related to cannabis. The Canadian model differs from other jurisdictions that legalized recreational cannabis use, especially with regard to a higher degree of government regulation of the cannabis market. Another difference is the development and endorsement of lower-risk cannabis use guidelines to educate the public and health professionals. The results available for this review cover only 3 years post-legalization. Cannabis legalization in Canada brought an apparent increase in use among Canadian older than 25. However, results for youth are mixed, with the majority of studies showing no pronounced increase. Notably, the trend of a decrease in adolescents' cannabis use seen pre-legalization may have reversed. Emerging evidence also suggests that cannabis-related hospitalizations and emergency department visits among Canadian youth may have increased due to cannabis legalization. Data about changes in the age of initiation, the influence of legalization on sex and gender, and race/ethnicity are limited, with evidence suggesting that the age of initiation slightly increased. So far, there is limited data about the impact of cannabis legalization on Canadian youth. Further long-term monitoring and research to assess the effects of cannabis legalization on Canadian youth.

## Introduction

Cannabis is one of the most globally prevalent psychoactive substances (1), with the onset of use usually in mid-adolescence (2, 3). Cannabis use has been linked to many short- and long-term adverse effects (4) including motor vehicle accidents (5), respiratory diseases (6), higher risk for acute myocardial infarction among people aged 15–22 (7), and cannabis use disorder (4, 8). In addition, youth who use cannabis are at increased risk for adverse mental health and cognitive outcomes, including development and exacerbation of early-onset psychosis (9), depression and anxiety (10), suicidal ideations and suicide attempts (11), alteration of brain development and structure (12, 13) lower educational attainment (14, 15), lower cognitive function, and decreased motivation (13). Also, individuals using cannabis during youth are at increased risk for addictive behaviors later in life (4).

The production, distribution, and consumption of non-medical cannabis were legalized in Canada at the federal (national) level in October 2018 (16). The federal government determines the types of cannabis products that can be legally sold. At the time of legalization, dried or fresh cannabis, cannabis oil, and cannabis seeds were the legal products. Edibles, extracts and topicals became legal 1 year later, and drinks 2 years after that (17).

Unsurprisingly, proposals for cannabis legalization for non-medical purposes (subsequently referred to as “cannabis legalization” or “legalization”) were controversial in Canada, especially in the context of protecting youth. The federal government stated that it was taking a public health approach, and that the *Cannabis Act* had three main goals: to “keep cannabis out of the hands of youth, keep profits out of the pockets of criminals, [and] protect public health and safety by allowing adults access to legal cannabis” (16, 18). The *Canadian Medical Association Journal* published an editorial in 2017 stating that the proposed legislation would fail to protect Canadian youth (19), to which others responded that prohibition was also harmful to youth and that legalization would provide the opportunity for strict regulation that would reduce cannabis-related harms (20, 21). In general, Canada's public health and substance use sectors were in favor of cannabis legalization for non-medical purposes (22, 23) while its medical community tended to be against it (24). As part of its approach to cannabis legalization, the federal government endorsed Canada's lower-risk cannabis use guidelines (25), which were designed as an evidence-based means of educating the public and health care professionals to reduce cannabis-related harms (25, 26).

This narrative review will focus on the impact of cannabis legalization on youth in Canada. This review aims to provide a broad description of the Canadian experience that may be of interest for jurisdictions considering the legalization of cannabis for non-medical purposes. We will describe the regulation of cannabis legalization for non-medical purposes and changes related to cannabis use and cannabis-related behaviors. Specifically, we will look at the prevalence of cannabis use, alternative modes of consumption, the potency of cannabis, age of first use, negative consequences related to use, and education regarding cannabis. This paper will use the World Health Organization's youth definition of ages 15–24 (27).

## Methods

Information used to write this narrative review was collected from the sources listed in Table 1.

**Table 1:** Information sources used for this narrative review.

• Electronic databases searches (e.g., Google scholar) for relevant journal articles • Internet hands searches of the references of the retrieved literature (e.g., policy reports, periodical surveys, governmental websites) • Professional experience in writing several documents on cannabis legalization for recreational purposes in Canada • Discussion with experts in the field of cannabis legalization for recreational purposes in Canada

## Cannabis regulation in Canada

Cannabis legalization in Canada took place with the passage of the *Cannabis Act* (16). While the *Cannabis Act* applies to the entire country, due to the nature of Canada's political system, some aspects of cannabis regulation have been set by the federal government and others by the governments of Canada's 10 provinces and three territories. Here we will discuss the elements of Canada's cannabis regulations most relevant to youth.

### Minimum age

The *Cannabis Act* set the minimum age for legal cannabis purchases at 18, with the provision that provinces and territories could raise (but not lower) it if desired (16). All but one opted to harmonize the minimum age for cannabis and alcohol, resulting in a minimum age of 19 everywhere except Alberta and Québec, where it was left at 18; later, Québec raised the minimum age for cannabis to 21. Notably, Québec's provincial public health institute opposed the increase in the minimum age, recommending instead that youth aged 18–20 be allowed to purchase only lower-risk products (maximum 10% THC) and smaller amounts (maximum 10 g of dried cannabis) (28).

The Act also states that underage individuals cannot legally possess more than 5 g of cannabis—in other words, youth under 18 cannot legally acquire cannabis but technically can possess under 5 g without legal consequences. This measure is “ostensibly in place to ensure that youth are not arrested for possessing small amounts of the drug” (29). Minors possessing over that amount may be charged under the *Youth Criminal Justice Act*, which prioritizes extra-judicial measures in order to avoid criminalizing youth (30). Provinces have also implemented their own penalties for underage possession of cannabis; generally, it is treated like underage possession of alcohol, punishable by a fine (31). This is further discussed below.

The *Cannabis Act* also raised the penalties for adults providing cannabis to a person under the age of 18 and for “using a youth to commit a cannabis-related offence,” both to a maximum of 14 years imprisonment (16).

### Possession and consumption

The *Cannabis Act* set upper limits on personal possession of cannabis by adults, with provinces and territories able to lower those limits if desired. Individuals above the minimum age can hold a maximum of “30 g of legal cannabis, dried or equivalent in non-dried form” in public (16). They can also grow up to four cannabis plants at home. However, two provinces (British Columbia and Québec) also placed limits on the amount of cannabis that can be possessed by a household (1 kg and 150 mg, respectively), and two provinces (Manitoba and Québec) banned home cultivation entirely (32).

Regulation of public cannabis use is left to provinces and territories (16). All limit the public smoking or vaping of cannabis in some way. Seven provinces and territories ban public cannabis smoking and vaping altogether. The other six allow cannabis smoking and vaping only in locations where tobacco smoking and vaping are allowed (32).

### Retail sales

Regulations in this area are determined by provinces and territories. Five have adopted public retail systems, in which government-owned entities are responsible for cannabis sales, online and/or in storefronts [several jurisdictions in Canada have this type of system in place for alcohol sales (33)]. Two have adopted private retail systems, in which sales are left to the private sector. Six have hybrid public/private (but private-dominant) systems, typically with a government entity responsible for online sales and in-person retail left to the private sector (32). The availability of legal cannabis varies accordingly: jurisdictions with private retail systems tend to have far higher numbers of stores per capita than those with public retail (34, 35).

Provinces and territories also differ with respect to limitations on retail locations (32):

- Five jurisdictions have a requirement that cannabis stores maintain a certain distance from schools—generally 150 m. Three allow communities to determine such requirements for themselves. Of note, the remaining five jurisdictions with no formal limitations have public retail systems, meaning that those provincial/territorial governments have direct control over where cannabis stores will be established.
- Eight jurisdictions allowed municipalities to opt out of hosting cannabis stores. We are not aware of any reviews of municipal cannabis store bans across Canada. However, Ontario, Canada's largest province by population, gave municipalities a one-time opportunity to opt out of having cannabis stores within their boundaries, and 19% (77 of 414) chose to do so—though 10 municipalities later reversed that decision (36). As a result, currently, about 18% of Ontario's population lives in municipalities where cannabis stores are not allowed and legal cannabis must be purchased online or by travelling to a municipality where stores are permitted.
- Significantly from a public health perspective, no province or territory has a formal limit or cap on the overall number of cannabis retail locations.

The packaging and labeling requirements described below apply to all cannabis products; the *Cannabis Act* also prohibits products that are appealing to youth (16). Further, cannabis edibles, extracts, and topicals are subject to THC limits: edibles, for instance, may contain no more than 10 mg of THC per package (37). For comparison, the state of Colorado sets a maximum potency for cannabis edibles of 10 mg of THC per serving, but allows up to 100 mg of THC per package (38). In Canada, illegal edibles seem to contain more THC than legal ones, with one study finding illegal products with over 150 mg of THC (39).

Legal sources of cannabis are slowly replacing illicit ones. The percentage of people who use cannabis aged 15+ who reported obtaining at least some of their cannabis from legal sources rose from 23% in 2018 to 47% in 2019 and 68% in 2020 (40).

### Advertising, marketing and promotion

**Figure 1:**
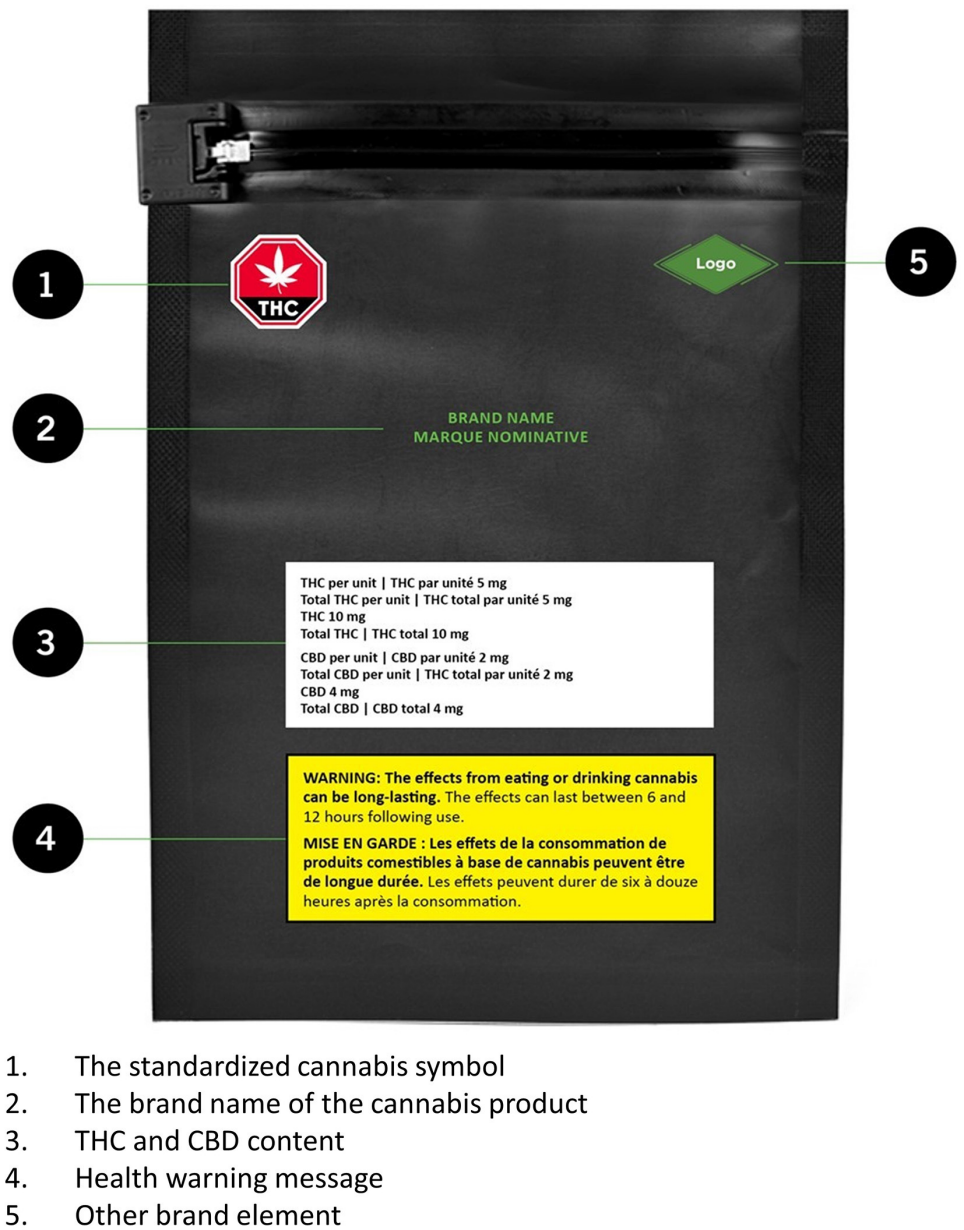
The anterior side of cannabis packaging in Canada. Source: ©All rights reserved. Packaging and Labelling Requirements. Health Canada, modified 2019. Adapted and reproduced with permission from the Minister of Health, 2021.

**Figure 2:**
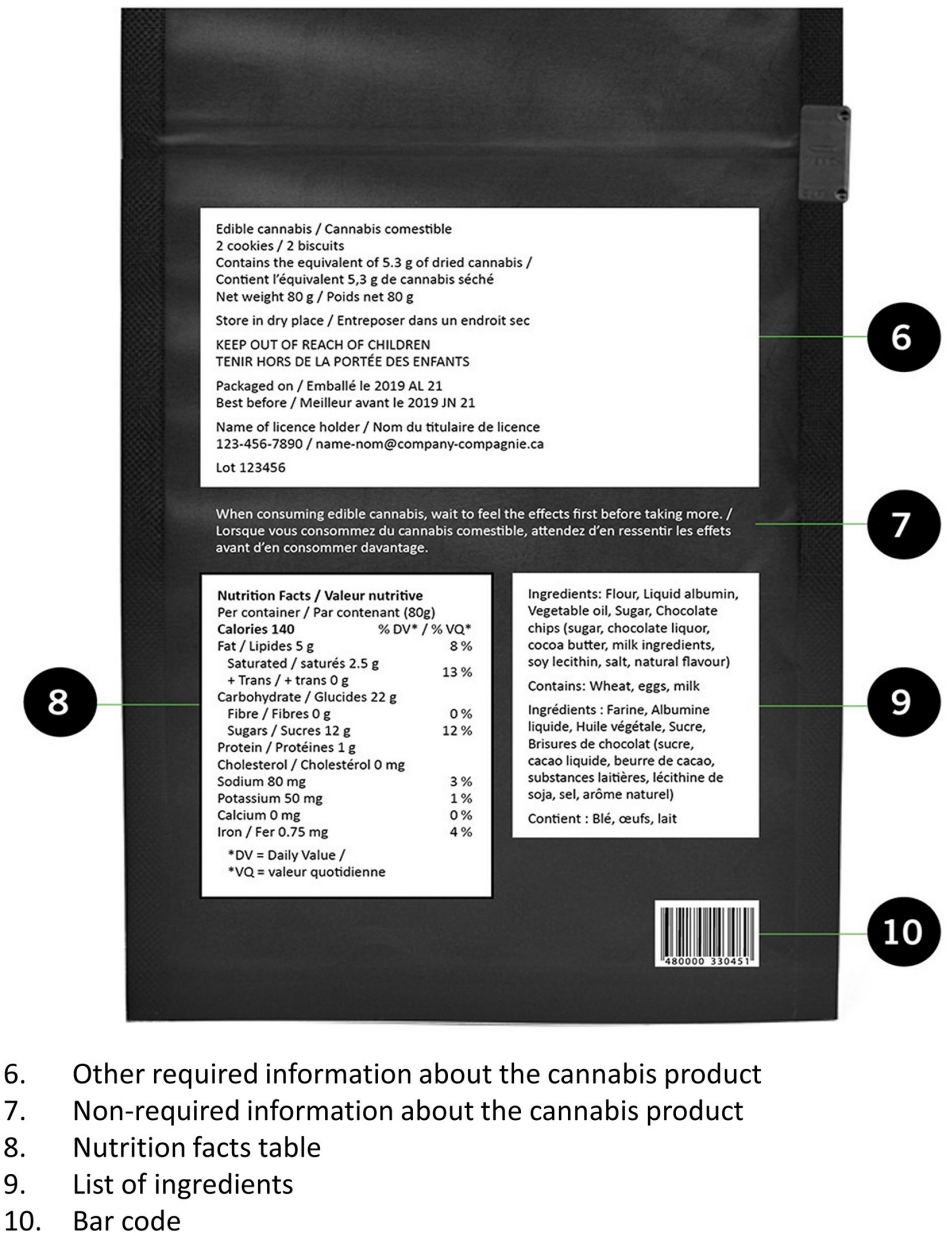
The posterior side of cannabis packaging in Canada. Source: ©All rights reserved. Packaging and Labelling Requirements. Health Canada, modified 2019. Adapted and reproduced with permission from the Minister of Health, 2021.

The *Cannabis Act* determines regulations in this area and they apply to the whole country. In addition to age restrictions, the federal government cites rules in this area as key to “protecting youth” (16). Advertising rules are quite strict: advertising is essentially banned outside of the point of purchase (i.e., cannabis stores) and celebrity endorsements are not allowed (37). In addition, cannabis products must be sold in plain packaging with standardized fonts (see Figures 1, 2 below); minimal brand elements are allowed. There is certain required information, including THC and CBD content and a standard health warning.

### In summary

Since the federal government introduced the *Cannabis Act* in 2016, it has continually emphasized the protection of youth as its main reason for cannabis legalization (36). Its stated rationale is that prohibition failed to curtail youth cannabis consumption, and legalization would provide the opportunity for cannabis to be strictly regulated (41). However, in promoting the *Cannabis Act*, the federal government went further, stating on numerous occasions that cannabis legalization would “keep cannabis out of the hands of kids” (36). While this claim was widely questioned [see (36) for a discussion], this priority of protecting youth is reflected in the legislation itself, which states at the outset that its purpose is to “protect the health of young persons by restricting their access to cannabis; [and] protect young persons and others from inducements to use cannabis” (37). As discussed below, so far there is limited data about the impact of cannabis legalization on Canadian youth. For now, we can simply point out that Canada's regulatory model looks very different from that of other jurisdictions with legal cannabis, especially those in the United States.

The most apparent difference between Canadian and American cannabis regulation is in the area of advertising and promotion: no US state has a ban on advertising or a requirement that cannabis be sold in plain, standardized packaging [due to constitutional protection of “commercial speech,” such regulations may not be possible in the US (42)]. There is emerging evidence that Canada's plain packaging makes cannabis less appealing to youth (43). Since there is strong evidence that youth are particularly susceptible to tobacco and alcohol advertising (44, 45), the ban on cannabis advertising is also noteworthy. Both of these regulations are among the measures recommended to the federal government by experts in the public health and substance use sectors (22, 46). However, violations of advertising rules by licensed cannabis firms seem widespread on the Internet, especially on social media (47); in addition, the cannabis industry has been lobbying for the ability to advertise and for plain packaging to be eliminated (48).

## Cannabis use prevalence among Canadian youth

This section will discuss trends in youth cannabis use since Canadian cannabis legalization. In Canada, a number of periodic surveys track the prevalence of cannabis use. We will present the available results from the surveys including individuals below 18 years old. Two of the surveys are specifically cannabis-related: The National Cannabis Survey (NCS) and The Canadian Cannabis Survey (CCS). The main aim of both surveys is to evaluate the impact of the *Cannabis Act* on cannabis-related behaviors/approaches (49, 50). The CCS is an online cross-sectional survey that has been conducted annually since 2017 with participants 16 and above (49). The NCS is another cross-sectional survey that has been conducted quarterly since 2018 (40). Other surveys that will be discussed do not focus primarily on cannabis. The Canadian Student Tobacco, Alcohol and Drugs Survey (CSTADS), is a cross-sectional survey, sampling students from grades 7–12 every 2 years (51, 52). The Cannabis use, Obesity, Mental health, Physical activity, Alcohol use, Smoking, and Sedentary behavior (COMPASS) is a survey with both cross-sectional and longitudinal data of high school students from four provinces in Canada (53, 54). And the Tobacco and Vaping Survey, which is a multi-wave survey conducted in Canada, England, and the United States (55). Table 2 presents the prevalence of cannabis use in the Canadian youth population before and after legalization by the different surveys; when applicable, the rate of daily or almost daily use is presented. We included only studies that presented pre- and post-legalization (2017–2021) data.

**Table 2:** The prevalence of Cannabis use in the Canadian youth population before and after legalization.

**Survey name**	**Age group**	**Time period**	**Prevalence of use (%)^mathbfe^**	**Prevalence of daily/almost daily use (%)^f^**	**1-6 times per week**	**Regular use**
Canadian Cannabis Survey	16–19	2017	41	N.A.	N.A	N.A
(49, 56–59)		2018	36	N.A	N.A	N.A
		2019	44	N.A	N.A	N.A
		2020	44	21	N.A	N.A
		2021	37	20	N.A	N.A
	20–24	2017	45	N.A	N.A	N.A
		2018	44	N.A	N.A	N.A
		2019	51	N.A	N.A	N.A
		2020	52	26	N.A	N.A
		2021	49	29	N.A	N.A
National Cannabis Survey (40)	15–17	2018 Q1	^a^	^a^	N.A	N.A
		2019 Q1	^a^	^a^	N.A	N.A
		2020 Q4	19.2^b^	^a^	N.A	N.A
	18–24	2018 Q1	28.1	9.9^b^	N.A	N.A
		2019 Q1	34.3	12.5^b^	N.A	N.A
		2020 Q4	35.6	16.3^b^	N.A	N.A
Canadian Student Tobacco, Alcohol, and	Grade 6–12 students	2014–15	17	N.A	N.A	N.A
Drugs Survey (60)	Grade 7–12 students	2016–17	17	N.A	N.A	N.A
		2018–19 (October 2018 and June 2019)	18	N.A	N.A	N.A
Cannabis use, Obesity, Mental health,	Grade 9–12 students	2016–17	15.9	3.9	5.5	9.4
Physical activity, Alcohol use, Smoking, and		2017–18^c^	16.4	3.9	5.6	9.5
Sedentary behavior (COMPASS)^g^ (53)		2018–19^d^	16.9	3.7	6.1	9.7
Tobacco and Vaping Survey (55)	16–19	2017	22.5	12.7	N.A	N.A
		2018^c^	23.4	13.7	N.A	N.A
		2019	27	17.6	N.A	N.A

^a^Unreliable to be published; ^b^use with caution; ^c^pre-legalization; ^d^post-legalization; ^e^all the results represent past-year prevalence, besides the National Cannabis survey in which results present prevalence of past- 3 months use; ^f^for COMPASS study and the Tobacco and Vaping Survey the results present current daily use; Q1, The first quarter; Q4, The fourth quarter; N.A, Not available.

### Before cannabis legalization for non-medical purposes

Canada has one of the highest youth cannabis use rates in the world (61, 62), and the prevalence of use among Canadian youth is about double the prevalence of use among people 25 years and older use (49). Nonetheless, data from pre-legalization years show a decreasing trend in adolescents' cannabis use (63–65).

### After cannabis legalization for non-medical purposes

A 2018 Canadian survey found that a substantial proportion of Canadian youth intended to try cannabis for the first time or increase their use following cannabis legalization (66). Accordingly, data collected in the CCHS throughout 2018 (67) show that use prevalence has increased to 16.3%, compared to 10.7% in 2017 among those aged 12–24 years old (64). CCS data also report an increase in the prevalence of past-year use between 2018 and 2020. In 2018, 36% of Canadian aged 16–19 years old endorsed cannabis use. In 2019 and 2021 this figure increased to 44% (56–58). This survey also showed an increase in cannabis use among Canadian aged 20–24, with the prevalence of increased from 44% in 2018 to 51% in 2019 and 52% in 2020 (56–58). Results from waves 1–3 of the Youth Tobacco and Vaping Survey also found that cannabis use prevalence increased significantly between 2017 and 2019 (55). This study also showed a significant increase in daily use [adjusted odds ratio (AOR) = 2.49] (55).

However, other data collected after legalization show no marked change in youth cannabis use. Results from the 2020 NCS report an increase in past-3 months use and daily use among 18- to 24-year-old Canadians compared to 2018 (pre-legalization) and 2019; however, the change is not statistically significant (40). Similarly, cross-sectional data from the COMPASS survey suggests an unremarkable increase for “ever use” (AOR = 1.05) in 2018–19 compared to 2016–2017 among Canadian high-school students, while no significant change was recorded in current and regular use. Longitudinal data from this study suggest that the differences between pre and post-legalization cohorts are minor (53). Data from the 2018 CSTADS found no change in use prevalence among students in grades 7–12 compared to 2016–17 (60).

Importantly, the most recent data from the 2021 CCS show a statistically significant decrease in youth cannabis use compared to 2020, with 37 and 49% of participants aged 16–19 and 20–24 (accordingly) reporting past year cannabis use, compared to 44 and 52% in the same age groups in 2020 (59). Unfortunately, so far, there are no other available survey results representing cannabis use in 2021 or 2022.

In summary, there are mixed results related to youth pre- and post-legalization cannabis use. Most studies show no pronounced/statistically significant increase; a few found an increase in youth cannabis use, and the most recent 2021 CCS, found a decrease in youth cannabis use. There is contradictory evidence regarding regular and daily or almost daily use, with some results suggesting no pronounced change and others suggesting an increase in youth daily use. Importantly, pre-legalization, Canada witnessed a trend of a decrease in adolescents' cannabis use. The initial evidence from the 2 years post-legalization suggests a possible reversal of that trend; however, the most recent study representing 2021 shows a decrease in cannabis use among both adolescents and emerging adults. Overall, results should be taken with caution as they are mixed and represent only up to 3 years post-legalization. This is important as a previous cross-national study suggests that the link between youth cannabis use and cannabis liberalization may only be statistically significant 5 years post- liberalization (68). Additionally, the surveys which target high school students might represent an underestimate since young who dropped out of school have higher rates of cannabis use (69).

### The effect of the COVID-19 pandemic

In March 2020, following the onset of the COVID-19 pandemic, Canadian provinces enacted new public health measures, including social distancing and closures of in-person schools (including high schools, colleges and universities) (70). Given the interference of those changes in daily routines, it was speculated that a shift in cannabis-related behaviors would be seen. So far, studies conducted in this context only evaluated the early and mid-stages of the COVID-19 pandemic. Their results are presented below.

One study found that fewer Canadians aged 16–18 reported cannabis use after social distancing practices started, but those who used reported increased frequency of use (71). Another study found that cannabis use among high school students increased between 2019 and 2020 (after the start of social distancing practices). However, this increase was smaller compared to the 2018–2019 period. In this study, the youth already using cannabis did not increase their use (72). Cross-sectional repeated data from the CCS 2020 and 2021 show that in 2020 many people who use cannabis have either increased or decreased in 2020 (56). However, in 2021 more people who use cannabis have increased their cannabis use in both age groups: 16–19 and 20–24 years old (59).

Interestingly, in a longitudinal study covering 4 months pre-pandemic and 3 months post-pandemic, participants aged 19–25 years who followed the recommendation to self-isolate were using 20% more cannabis than those who did not follow self-isolation recommendations. Using cannabis to cope with depression predicted an increased quantity and frequency of cannabis use during the pandemic (73).

In summary, both increases and decreases in cannabis use were observed in the early stages of the COVID-19 pandemic. However, more recent data suggest an overall increase in cannabis use among Canadian youth. Self-isolation and using cannabis to cope with depression were associated with increased cannabis use. Also, the majority of the available data represent the early period of the pandemic, and there is a need for evidence representing later and post-pandemic periods and also evidence about vulnerable populations, such as people that deal with mental health disorders.

### Cannabis potency

Cannabis potency is typically defined as the percentage of delta-9-Tetrahydrocannabinol (THC) (the main psychoactive component) in the cannabis product (74). In the past decades, there has been a strong increase in cannabis potency (4, 75–77). For example, in the United States, the mean potency of cannabis plant material increased from 8.9% THC in 2008 to 17.1% THC in 2017 (76). This trend is concerning, as higher-potency cannabis is associated with more short and long-term health risks (74, 78). While THC levels have increased over time, cannabidiol (CBD) levels have not substantially changed (77) CBD is another major component of cannabis. Evidence suggests that CBD is well-tolerated and has relatively very few serious adverse events, such as drug-to-drug interactions, pneumonia, hepatic abnormalities, diarrhea, fatigue, vomiting and somnolence (79, 80). CBD is correlated with several positive effects, such as neuroprotective (81), anxiolytic (82), and antipsychotic (83) effects. Of note, with higher THC to CBD ratios, there is a potential to exacerbate the adverse effects of THC [see (84) for a review].

CCS cross-sectional data show that in 2020, 35.5% of people who use cannabis between the ages of 16–19 reported using strains with high THC, as did 32.9% of people who use cannabis aged 20–24. 5.1% of the 20–24 years old group used only THC (unfortunately, data about the use of only THC is unavailable for the younger age group in 2020) (85). The 2021 CCS showed that around one-third of people who use cannabis ages 16–19 and 20–124 reported using cannabis strains with higher THC and lower CBD and around 8% used strains with only THC (85). Another study that tracked the potency of legal and illegal cannabis products for 2 months following legalization in Canada found a mean THC concentration of 16.1% in the legal market and 20.5% in the illegal market (86). These studies both took place after legalization; to the best of our knowledge, no published study has assessed changes in cannabis potency in Canada before and after legalization.

In summary, data suggest that in the last few years, higher-potency cannabis has gained popularity; however, there is no available data about changes in potency before and after legalization. Research is needed to monitor future changes in this area.

### Changes in modes of cannabis use

Data from the US suggest that modes of cannabis use other than smoking (e.g., vaping and edibles) increased in popularity along with the liberalization of cannabis (87). This finding is important, as edible cannabis products tend to have higher potency and delayed effects compared to smokable products, and thus may lead to over-intoxication (88, 89). Also, when removed from their packaging, they may look like food, thus increasing the risk for unintentional consumption, especially by children (89). Vaping cannabis is also a concern. Although it exposes the individual to fewer toxins compared to smokable cannabis, it has been associated with cases of acute lung injury and often involves high-potency cannabis products, which increases long and short-term risks (90). Potent cannabis extracts also increase the risk of over-intoxication, and limited evidence suggests that frequent extract use is linked with problematic cannabis use, cannabis use disorder and mental health problems (89). The long-term health impact of these alternative modes of cannabis use is unknown (89).

One study suggests that smoking was still the most prevalent mode of use among Canadians aged 16–19, with no significant changes between 2017 and 2019. However, in those years, e-cigarettes and extracts have gained popularity (AOR for e-cigarette use was 2.39 and the AOR for extract use 1.61 in 2019 compared to 2017) (55). Data from COMPASS show that 42.4% of Canadian high school students expanded their modes of use in 2018–19 compared to 2017–18. High school students who consumed cannabis in multiple modes were significantly more likely to binge drink alcohol, vape, use cannabis regularly, and endorse experiencing more depressive symptoms (53).

In summary, data suggest that after cannabis legalization, the Canadian youth extended modes of cannabis use, including more potent forms of cannabis. However, there is no data regarding whether youth using cannabis are obtaining it from a legal or illegal source. This distinction is important, as legal edibles, extracts, and topical products are subject to THC limits.

## Legalization and youth cannabis use: Special considerations

In this section, we will discuss special considerations such as the age of initiation, the influence of sex and gender, as well as race and ethnicity.

### Age of initiation

Delaying the age of initiation of cannabis use is an important priority for public health, as early initiation of cannabis use (e.g., before age 18) has been clearly shown to increase the subsequent risk of cannabis dependence (91) and has the potential to lead to more severe and long-lasting cognitive and neurodevelopmental adverse effects (92, 93). Among youth aged 16–19 in the CCS, the mean age of initiation increased slightly from 2018 (15.2 years) to 2019 (15.6 years), with less of an increase to 2020 (15.7 years) that fell back to 15.6 years in 2021 (85, 94). These estimates are slightly higher than data from previous youth surveys have suggested. For example, Canadian youth data from 2004/05 to 2014/15 from the CSTADS found a mean age of initiation of cannabis use of 13.8 years, which did not vary significantly over the time period from 2004/2005 to 2014/2015 (65).

In summary, the limited data available suggest that the age of initiation of cannabis use might have increased slightly from pre- to post-legalization but has likely not decreased.

### Influence of sex and gender

Both sex and gender impact cannabis use and effects. Animal models have shown that females are more sensitive to the effects of THC and other cannabinoids and that gonadal hormones (e.g., estrogens) directly impact THC metabolism and endocannabinoid system signaling (95). Cannabis use and cannabis use disorder have a more significant effect on self-reported mental health quality of life among women than men (96). Human laboratory research has suggested that females experience the same subjective effects as males after smoking less cannabis, which recapitulates some of the sex differences in acute cannabinoid effects observed in animal models (97, 98). Human research has shown that gender significantly impacts access to cannabis and patterns of cannabis use, with men/boys significantly more likely to use cannabis than women/girls (99). For example, the 2018 CCS found that 26% of men reported past-year use of cannabis compared to 18% of women (100). A recent analysis of federal data in Canada has suggested that men/boys might be increasing their use of cannabis to a greater extent than women/girls. Using data from the 2018 (pre-legalization) and 2019 (post-legalization) cycles of NCS found an increase in past-3-month cannabis use from 17.5 to 20.3% in men/boys aged 15+, compared to an increase from 12.3 to 13.4% among women/girls aged 15+. However, in the 2020 NCS data, for the first time, there was no statistically significant difference in the prevalence of past-3-month cannabis use between men/boys (21.1%) and women/girls (18.4%) (40). The difference in past-year cannabis use was still significantly different by sex (31% for males, 23% for females) in the CCS data that included Canadians aged 16 years or older (94). Unfortunately, these data rarely provide a breakdown of gender differences by age group, so it is not clear how the legalization of cannabis might have impacted boys and girls differently. In addition, no data were located that included gender-diverse or non-binary youth. In the CCS data, the frequency of daily or near-daily cannabis use in the past 12 months was broken down by both sex and age, showing a greater sex difference in adults aged 25 or older (30% for males, 19% for females) than in young adults aged 20–24 years (26% for males, 20% for females) and in youth aged 16–19 years, where the prevalence of daily or near-daily use was actually higher in females (20% for males, 23% for females). In a study assessing youth seeking support in a concurrent disorder treatment program, the proportion of boys/men sourcing cannabis from dealers increased, while the proportion decreased among girls/women after legalization. There was no other significant gender-related change between pre and post-legalization (101).

In summary, it appears that the prevalence of cannabis use is becoming more similar among men/boys and women/girls, though it remains unclear to what extent this might be due to legalization, and the data are too sparse to know if this is specifically true in youth, or if this is a trend mainly observed in adults.

### Influence of race/ethnicity

Data that speak to racial/ethnic differences in cannabis use comparing pre-and post-legalization could not be located. As discussed by Haines-Saah and Fischer, the lack of racial/ethnic data on cannabis-related outcomes in Canada is a significant problem, as youth of color (especially Black and Indigenous youth) have been disproportionately targeted by policing and criminal justice systems in Canada (102). While not specifically focused on youth arrests, Owusu-Bempah and Luscombe recently showed that Black and Indigenous people in five major Canadian cities were significantly more likely to be arrested for cannabis possession than white people (103). How cannabis legalization has impacted youth of color in Canada and whether the disproportionate targeting and surveillance of Black and Indigenous youth in Canada have lessened with cannabis legalization is an important topic of further research. Starting in 2020, the CCS began to report past-12-month cannabis use by ethnicity, sex, and age group, though the data on youth were often suppressed due to high sampling variability or small sample size. Data broken down by Indigenous status showed a high prevalence of cannabis use among Indigenous youth aged 16–24 years—where 62.3% of First Nations (North American Indian) youth reported past-12-month use, 56.1% of Métis youth reported past-12-month use (the Métis people are Indigenous peoples native to the provinces of Manitoba, Saskatchewan, Alberta, parts of Ontario, British Columbia, and the northern United States), while 48.3% of youth who did not identify as Indigenous reported past-12-month use (94). Comparable data from the 2021 CCS were not available due to high sampling variability or low sample size. When data were broken down by ethnicity, only data on white youth in the 16–19 year age group were available (49.2% reported past-12-month use), though data on other ethnicities were available for young adults aged 20–24. In this age group, 58.8% of white young adults reported past-12-month use, compared to 36.2% of South Asian young adults, 40.3% of Black young adults, 47.7% of Latin American young adults, and 53.1% for young adults who fell into “Other” ethnicity. In the 2021 CCS, 56.3% of white young adults reported past-12-month use, 32.9% of South Asian youth, and 29.1% of East/Southeast Asian youth, while data from other race categories were not available (85).

In summary, more data are needed to clarify how cannabis legalization might have impacted youth of color in Canada.

### Impact of cannabis legalization for non-medical purposes on other cannabis-related outcomes among youth

While there has been substantial focus on the impact of cannabis legalization on cannabis use prevalence and frequency among youth, considerably less attention has been paid to cannabis-related harms among youth.

Emerging evidence suggests that cannabis-related hospitalizations and emergency department (ED) visits among Canadian youth may have increased due to cannabis legalization. Using data that was collected during the initial 5–6 months following cannabis legalization, two studies evaluated the impact of legalization of cannabis in Canada on youth cannabis-related hospitalizations, defined in both studies as admissions for poisonings or for mental or behavioral effects of cannabis using the 10th revision of the International Classification of Diseases (ICD-10). The first study (conducted in the province of Quebec) found no significant evidence of an increase in cannabis-related hospitalizations among youth, comparing a pre-legalization period (October 17, 2017–March 31, 2018) to a post-legalization period (October 17, 2018–March 31, 2019). However, in boys aged 10–14 years, there was a significant increase in the proportion of substance-related hospitalizations involving cannabis, from 39.3% pre-legalization to 70.0% post-legalization (104). The second study, a retrospective chart review of cannabis-related visits to an academic ED in Hamilton, Ontario, did find a significant 56% increase in ED visits following cannabis legalization among young adults aged 18–29 years (105). A recent repeated cross-sectional study conducted in Ontario evaluated ED visits and related hospitalizations involving cannabis exposure covering a long period pre- and post-legalization (between January 2016 and March 2021) (106). This study identified a total of 522 ED visits due to cannabis exposure over the study period; 81 visits occurred during the pre-legalization period (January 2016–September 2018), 124 visits occurred during the period immediately after the legalization of cannabis flower products (October 2018–January 2020), and 317 visits occurred during the period after commercial sale of cannabis edibles was legalized (February 2020–March 2021), which represented a significant pre-post increase for both post-legalization time periods. Importantly, even after adjusting for the increasing time trend of cannabis-related hospitalizations over the study period, the period following the legalization of commercial sale of cannabis edibles remained significantly associated with an increase in ED visits (106). Another study conducted in the province of Alberta found that while there was not an overall increase in the volume of pediatric cannabis-related ED visits in a post-legalization period (October 1, 2018–March 1, 2020) compared to a pre-legalization period (October 1, 2013–September 30, 2018), there was a significant increase in unintentional ingestions of cannabis among both children and older adolescents (107). Finally, a retrospective chart review of ED visits at a pediatric hospital in Ottawa, Ontario found an increase in pediatric ED visits related to unintentional exposures to cannabis, though the absolute number was still relatively low (5 visits in the 5-year period prior to legalization, 32 visits in the 2-year period following legalization) (108).

Data on cannabis-involved driving among youth are limited and mixed. Preliminary results from the NCS suggested that the percentage of young adults (aged 18–24 years) reporting driving within 2 h of using cannabis in the past 3 months actually decreased following legalization, from 16.4% pre- to 9.7% post-legalization. Being a passenger in a vehicle operated by a driver who had consumed cannabis within 2 h of driving in the past 3 months similarly decreased from 15.2% pre- to 11.9% post-legalization. CCS data are somewhat harder to compare, as cannabis-involved driving questions changed slightly with each new year of data. In 2018, 26.7% of youth aged 16–19 who used cannabis in the past 12 months reported having driven within 2 h of using cannabis, compared to 16.0% who reported having driven within 2 h of smoking or vaping cannabis in 2019, which was nearly identical in 2020 (16.1%) (94). In support of these findings, a recent study found no significant association between cannabis legalization and traffic injury ED visits among youth drivers (aged 14–17 years in the province of Alberta, 16–18 years in the province of Ontario) (109). However, a recent study utilizing data collected from four trauma centers in the Canadian province of British Columbia between January 2013 and March 2020 indicated an increased prevalence of injured drivers under age 30 with a THC concentration of at least 2 ng/mL in blood, which is a threshold to define cannabis-impaired driving in some jurisdictions (110).

Cannabis legalization has the potential to impact youth seeking mental health support. A recent study analyzed data from one pre-legalization cohort and one post-legalization cohort of youth accessing treatment through an outpatient addictions and concurrent disorders treatment program for youth offered by the Centre for Addiction and Mental Health in Toronto, Ontario. They found no significant differences in a range of cannabis-related outcomes associated with legalization, including no changes in youth polysubstance use and no changes in mental health or substance dependence symptoms (101). Using a similar retrospective observational design, another study examined data from patients at least 12 years old who had visited a psychiatrist in the emergency unit of the Centre hospitalier universitaire de Sherbrooke (CHUS), comparing a period of 5 months post-legalization to a period of 2 years prior to legalization. They observed a significant increase in diagnoses of a cannabis use disorder, which was especially prominent among patients aged 18–24 years (from 17.3% pre-legalization to 25.9% post-legalization), though there was no significant increase in diagnoses of a psychotic disorder. This study also reported a significant increase in active use of cannabis among young adults presenting for psychiatric services, from 37.9% pre-legalization to 52.3% post-legalization. These data suggest that extra care should be taken to screen for cannabis use and potential cannabis use disorder among youth presenting for psychiatric services (111).

In contrast to potential harms associated with legalization, recent evidence about legal encounters suggests a possible net benefit to youth. Using data from the Canadian Uniform Crime Reporting Survey (UCR-2), a recent study found that implementation of the *Cannabis Act* was associated with a significant decrease in police-reported cannabis-related criminal offences among youth aged 12–17 years, an effect that was significant among both boys and girls. Importantly, no association was observed between the implementation of the *Cannabis Act* and property crimes or violent crimes among youth, providing preliminary evidence that cannabis legalization was not associated with overall increases in youth crimes (112). Data reported by the Canadian Centre for Justice Statistics similarly found a decrease of 36% in cannabis possession cases among youth (aged 12–17 years) from 2017 to 2018 (113). Finally, a recent report using data from Statistics Canada found a dramatic reduction in the number of cannabis possession charge counts among youth aged 12–17 years in Canada, from 9,383 cases in 2017 to just 740 cases in 2019 (114).

In summary, data on changes in cannabis-attributable harms associated with cannabis legalization among youth are limited and mixed. Though very preliminary, the current data do suggest an increase in cannabis-related hospitalizations among Canadian youth, which is possibly related specifically to the legalization of commercial sale of edible cannabis products. Unlike in the United States (Colorado, Washington State), the limited data do not suggest any major changes in driving under the influence of cannabis among youth, though an increase in THC-positive drivers under age 30 that were involved in car accidents was noted in British Columbia. Data from one university health center in the province of Quebec found an increase in cannabis use disorder diagnoses among young adults immediately following legalization, but it is unclear whether this generalizes to the rest of the country. Encouragingly, cannabis legalization appears to have led to a significant decrease in cannabis-related criminal offences among youth, with no significant changes in property or violent crimes.

### Implications of legalization for education/prevention, treatment, and clinical practice

Health Canada committed CAD$100 million over 6 years to support education, awareness, and surveillance related to cannabis (115). Within the provinces and territories, the approach to prevent cannabis-related harms varies. So far, little data exists to determine if these approaches have been effective in reducing cannabis-related harms among youth or not. However, starting in 2019, the CCS began collecting data on exposure to and attitudes toward cannabis educational campaigns. In 2019, among youth aged 16–19 years, only 11.0% reported not noticing any cannabis-related education campaigns or public health messages. The most common locations for exposure to cannabis education campaigns or messages included social media (73.2%), school (58.1%), TV/radio (46.7%), or posters/billboards displayed publicly (46.7%). Also, in 2019, respondents were asked if their knowledge of cannabis-related harms increased since the new cannabis law came into effect; 30.9% of youth aged 16–19 years said “yes,” while 39.5% said “no,” and 25.4% said “somewhat.” In 2020, respondents who reported seeing educational messages were additionally asked about the perceived credibility of the education campaigns, public health or safety messages. Overall, 71.3% of youth aged 16–19 years found the messages to be credible, while 21.0% found the messages “somewhat” credible (94). Thus, it appears that youth in Canada are being exposed to cannabis-related public health messaging, and the messages are being perceived as credible and trustworthy, but further data are needed to evaluate if these messages are having a positive impact on the cannabis use behaviors of youth, especially in regards to cannabis-attributable harms.

At the level of the school, data from the COMPASS study have described disciplinary approaches to cannabis use policy violations in Canadian secondary schools (116, 117). While punitive options (e.g., suspension from school, alerting the police) were more common than supportive options (e.g., encouraging participation in a substance use education program), the results showed that schools were less likely to use alerting the police as a punitive option for students violating cannabis use policies in the year post-legalization compared to the year pre-legalization (116). Clearly, more resources are needed for schools and school boards to facilitate implementation of supportive approaches to cannabis use policy violations in Canadian high schools.

A few educational interventions targeting youth were identified that have been evaluated since cannabis legalization or are currently under evaluation. For example, the Check Your Cannabis brief intervention is an anonymous digital health brief intervention based on normative feedback, harm reduction, and motivational interviewing, which has been evaluated as a potential tool for cannabis-involved driving concerns among youth, but can be tailored to focus on other cannabis-related concerns (118). The use of low-cost digital health interventions may be particularly useful to target youth who experience more cannabis-related problems. Another example is the University of Calgary's *Cannabis Café*, a novel harm reduction educational initiative that follows Canada's Lower-Risk Cannabis Use Guidelines and scientific evidence and targets post-secondary students (119).

In summary, data suggest that Canadian youth are being exposed to cannabis-related health messaging, which is encouraging. However, it is unclear how effective these messages are. More data are needed to evaluate the impact of cannabis legalization in Canada on education and prevention.

## Conclusion

Cannabis legalization for non-medical purposes took place in Canada in October 2018. One of the federal government's stated goals in legalizing cannabis was to protect Canadian youth from cannabis-related harms (16). In this narrative review, exploring the impact of cannabis legalization on Canadian youth, we described the Canadian experience in detail. We discussed the regulation of the Canadian cannabis market, outlined changes in the epidemiology and parameters of cannabis use among youth, and discussed cannabis related prevention and education initiatives. The purpose of this review is to provide a broad description of the Canadian experience that may be of value to jurisdictions considering the legalization of cannabis.

The Canadian model differs from other jurisdictions which legalized recreational cannabis use in a number of aspects. First and foremost, the level of cannabis market regulation is much more extensive, with strict limits on packaging and labeling as well as advertising, marketing and promotion. Another difference is the government's endorsement of Canada's lower-risk cannabis use guidelines (25) as a way to educate the public and public health professionals (26).

Despite being legalized at the federal level, many rules are set at the provincial/territorial level, and thus impacts are likely to vary across the country. Although it is too early to ascertain, there are indications that Canadian provinces and territories with looser cannabis retail sales regulations have seen a higher increase in use compared to those with stricter regulations (120). This would be consistent with the alcohol and tobacco literature, which suggests that relative increases in availability are associated with increased consumption (33, 35). From a public health and youth protection perspective, provinces and territories should develop and enforce limits on retail density (35).

The *Cannabis Act* includes a requirement that the federal government review the legislation 3 years after it comes into effect. This review is expected to be conducted in 2022. According to the Act, the review must prioritize the impact of cannabis legalization on “public health and, in particular, on the health and consumption habits of young persons in respect of cannabis use” (24). The cannabis industry has been pushing for a repeal on many of the Act's regulations, notably the requirement for plain packaging and the restrictions on advertising, marketing and promotion (48, 121). It is critical that these regulations be maintained, from a public health perspective, as it is less appealing for youth compared to non-plain packaging (35).

While cannabis legalization brought an apparent increase in use among Canadians older than 25 years of age (40, 56), results for youth are mixed. Most studies show no pronounced increase, a few studies suggest an increase and the most recent national survey suggest a decline in youth cannabis use. Overall, however, the results suggest that the trend of a decrease in adolescents' cannabis use seen pre-legalization (64) may have reversed.

Data about changes in the age of initiation, the influence of legalization on sex and gender, and race/ethnicity are limited, with evidence suggesting that the age of initiation slightly. Also, data collected after legalization suggests that the prevalence of cannabis use among Canadian Indigenous youth is higher, compared to other ethnical groups (94). Notably, data on changes in cannabis-attributable harms among youth associated with legalization are limited and mixed, with emerging evidence suggesting an increase in youth hospitalization and ED visits as a result of cannabis legalization. Canadian youth are being exposed to cannabis-related health messaging, which is encouraging. However, it is unclear how effective these messages are.

The results presented in this review might support effective policies and educational initiatives. In particular, the finding that the trend of a decrease in adolescents' cannabis use has either reversed or stopped can further support educational endeavors. This finding can also be used by the public health sector advocating for continuing the strict packaging restrictions. In addition, the finding of increased cannabis use among indigenous youth, compared to other ethnical groups (94) stresses the need for prevention and treatment programs targeting specific groups. Thus far, there is limited evidence linking specific policies to public health consequences. Future research about it can be a useful resource for both Canada and other jurisdictions considering the legalization of cannabis for recreational purposes.

The results available for this review cover only 3 years post-legalization; thus, it is not surprising that the data is limited and mixed. Further monitoring and research are needed to assess the impact of cannabis legalization on Canadian youth and the current results should be taken with caution.

## Author contributions

DR-K, J-FC, JM, and BL contributed to the conceptualization and methodology. DR-K, J-FC, and JM contributed to writing the original draft preparation. All authors contributed to the review and editing, read, and approved the final manuscript.

## Funding

DR-K receives salary support from the O'Brien Scholars Program within the Child and Youth Mental Health Collaborative at CAMH and the Hospital for Sick Children, Toronto, Canada. JM received financial support from the Toronto Cannabis & Cannabinoid Research Consortium (TC3) and the Centre for Addiction and Mental Health (CAMH) womenmind community. BF is supported by CAMH, a clinician-scientist award from the Department of Family and Community Medicine of the University of Toronto and a Chair in Addiction Psychiatry from the Department of Psychiatry of University of Toronto and by Waypoint Centre for Mental Health Care.

## Conflict of interest

Author JM is supported by a Mitacs Accelerate award granted to BL in partnership with Canopy Growth Corporation. The remaining authors declare that the research was conducted in the absence of any commercial or financial relationships that could be construed as a potential conflict of interest.

## Publisher's note

All claims expressed in this article are solely those of the authors and do not necessarily represent those of their affiliated organizations, or those of the publisher, the editors and the reviewers. Any product that may be evaluated in this article, or claim that may be made by its manufacturer, is not guaranteed or endorsed by the publisher.
